# Codon Usage Bias Variation and Evolutionary Signatures of Epstein–Barr Virus in Distinct Epithelial Cancers

**DOI:** 10.3390/v18040425

**Published:** 2026-03-31

**Authors:** Xiaoqian Li, Xianyang Huang, Wan-Ting Li, Guy Baele, Liyuan Liu, Siyan Li, Jianjun Dai, Wan-Ting He

**Affiliations:** 1 School of Pharmacy, China Pharmaceutical University, Nanjing 211198, Chinajjdai@cpu.edu.cn (J.D.); 2 Department of Microbiology, Immunology and Transplantation, Rega Institute, KU Leuven, 3000 Leuven, Belgium; guy.baele@kuleuven.be

**Keywords:** Epstein–Barr virus, gastric cancers, nasopharyngeal carcinoma, codon usage pattern

## Abstract

EBV genomic variation has been shown to contribute to the development of certain EBV-associated cancers. While EBV genomic variation has been extensively studied at the nucleotide level, it remains unclear how synonymous codon usage contributes to viral adaptation across epithelial cancer contexts. Here, we analyzed 1148 EBV genomes with annotated tumor origins to investigate genome-wide genetic differentiation and codon usage patterns of 13 core genes across NPC- and GC-associated viruses and EBV types. SNP-based analyses revealed partial genetic separation between NPC-EBV and GC-EBV, characterized by both rare GC-associated risk variants and common protective haplotypes. Codon usage patterns, however, showed strong gene-specific structure: *EBNA2* and *EBNA3* clustered primarily by EBV type, whereas *EBNA1* and *LMP2A* were more sensitive to tumor background. Codon bias analyses suggested heterogeneous contributions of mutational pressure and natural selection across genes and lineages, whereas lytic *BALF* genes displayed highly conserved codon usage despite cancer-associated variants. Collectively, this study demonstrates that codon usage patterns of specific EBV genes are associated with tumor background and are jointly shaped by gene function and viral lineage structure.

## 1. Introduction

Infectious pathogens are strong and modifiable causes of cancer [1]. The Epstein–Barr virus (EBV)—discovered  as the first human tumor virus years ago by Anthony Epstein, Yvonne Barr and Bert Achong—is one of the most prominent pathogens in humans, persistently infecting more than 90% of the human adult population [2]. EBV contributes to about 1.5% of all cases of human cancer worldwide [3], and viral genes are expressed in the malignant cells. EBV also very efficiently causes the proliferation of infected human B lymphocytes [4]. EBV, also known as human herpesvirus 4 (HHV-4), is a member of the gammaherpesvirus family 4. EBV belongs to the gamma 1 or lymphocryptovirus genus and was identified as the first human tumor virus, which was later found to be associated with epithelial cancers including nasopharyngeal carcinoma (NPC) and a subset of gastric cancers (GC) [5,6]. There are known differences in phenotypic properties between EBV isolates. There are two types of EBV isolates—type 1 and type 2—which have sequence polymorphisms in the EBV nuclear antigen 2 (*EBNA2*) and EBV nuclear antigen 3 (*EBNA3*) genes [5]. The double-stranded DNA genome of EBV encodes a repertoire of genes that orchestrate both the virus’s life cycle and its interactions with host cells. Through large-scale genomic studies, two nonsynonymous EBV variants, *BALF2*, have been identified as being closely associated with the risk of nasopharyngeal carcinoma [7]. Recent studies have reported that the distribution patterns of EBV single nucleotide variations (SNVs) and structural variations (SVs), particularly large deletions, vary markedly across different diseases. Codon usage was found to differ between latent and lytic cycle EBV genes [8].

Codon usage bias has been analyzed in several viruses, for example in human cytomegalovirus (HCMV) [9] and pseudorabies virus [10]. Previous studies have successfully demonstrated that detailed analyses of codon usage patterns can provide theoretical insights into the evolutionary dynamics of HCMV [9]. Codon usage bias refers to the non-random selection and use of specific synonymous codons when encoding the same amino acid [10,11]. However, despite substantial progress in characterizing EBV genomic variation, current studies have largely focused on single-nucleotide variants and structural alterations, with comparatively little attention paid to codon usage patterns at the gene level. In particular, it remains unclear whether EBV adapts its translational strategies through differential codon usage in distinct epithelial malignancies, such as nasopharyngeal carcinoma and gastric cancer. Moreover, although EBV type 1 and type 2 strains exhibit well-established sequence polymorphisms and biological differences, systematic comparisons of codon usage bias between viral types across cancer contexts are lacking. Whether such variation is gene-specific, cancer-type-dependent, or reflects broader viral adaptation to epithelial cell environments has not been comprehensively investigated [12]. Here, we analyzed 1148 EBV genome sequences with clearly annotated tumor origins to systematically investigate the codon usage patterns of 13 EBV genes across EBV-associated epithelial malignancies, including nasopharyngeal carcinoma and gastric cancer, and to further compare differences between EBV type 1 and type 2 strains. By integrating cancer-type comparisons with viral genotyping, we aimed to characterize gene-specific and cancer- as well as lineage-dependent codon usage biases within the EBV genome. This integrative framework provides insight into how EBV translational strategies vary across tumor contexts and viral lineages.

## 2. Results

### 2.1. Distinct Genomic Structure and Cancer-Type–Associated Genetic Variants Between NPC-EBV and GC-EBV

A total of 1148 Epstein–Barr virus (EBV) genome sequences with clearly annotated cancer origins—nasopharyngeal carcinoma (NPC) and gastric carcinoma (GC)—were retrieved from the NCBI database [13,14]. Among these, 205 complete EBV genomes were retained for subsequent single nucleotide polymorphism (SNP) analyses (Supplementary Table S1). According to their cancer origins, these sequences were classified as NPC-associated EBV (NPC-EBV) and GC-associated EBV (GC-EBV), comprising 176 and 29 sequences, respectively.

**Figure 1:**
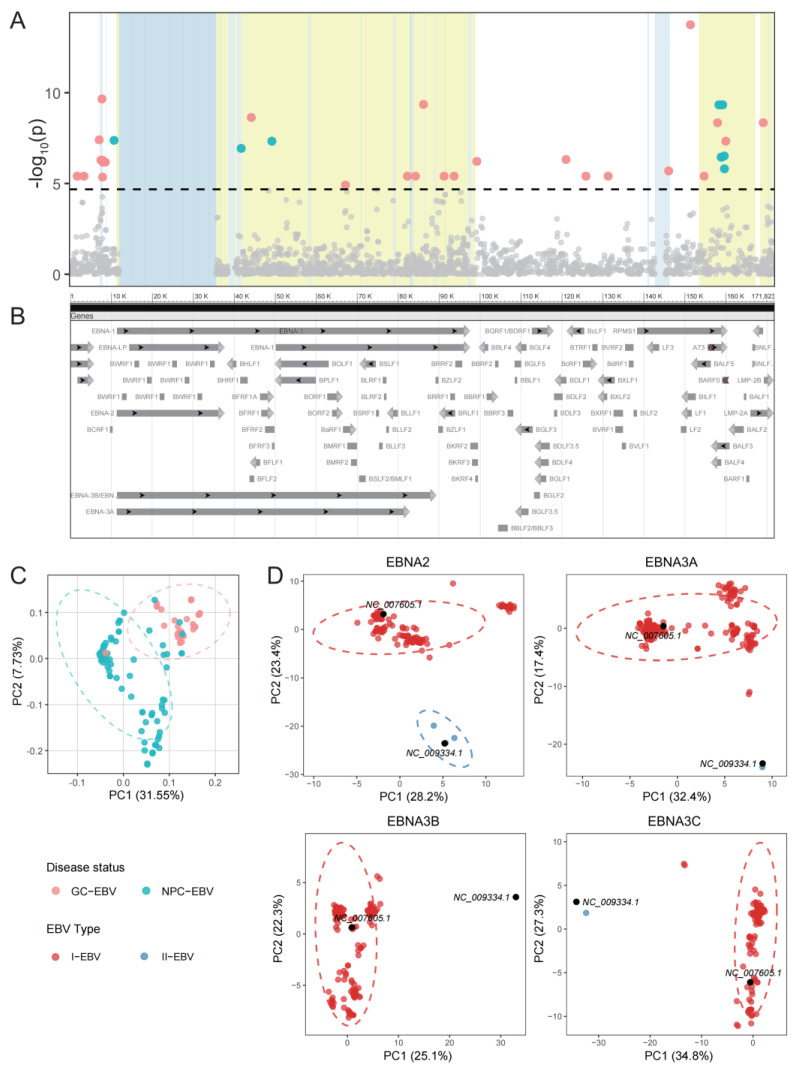
Genome-wide principal component analysis and association analysis of NPC-EBV and GC-EBV variants. (**A**) Manhattan plot of the genome-wide association analysis based on a linear mixed model (LMM). The *x*-axis represents the physical positions of variants along the EBV genome, and the *y*-axis shows the −log_10_(P) values for association between NPC-derived EBV isolates (*n* = 176) and GC-derived EBV isolates (*n* = 29). The red dashed line indicates the suggested genome-wide significance threshold (*p* = 2.09 × 10^−5^). SNPs exceeding this threshold are highlighted. Among them, red dots denote GC risk alleles (*β* > 0), whereas blue dots denote NPC risk alleles (*β* < 0). (**B**) Schematic representation of the EBV genome structure. Repeat regions are shaded in light blue in panel (**A**), and the *EBNA*, *LMP*, and *BALF* gene family regions are highlighted in light yellow. (**C**) Principal component analysis (PCA) based on 205 EBV whole-genome sequences, including 176 NPC-EBV and 29 GC-EBV genomes. The first two principal components (PC1 and PC2) are shown. PC1 explains 31.55% of the total genomic variation and effectively separates NPC-EBV from GC-EBV. Points are colored according to cancer background. Ellipses indicate 95% confidence intervals. (**D**) EBV genome sequences from the NCBI database with clearly annotated cancer backgrounds (NPC or GC) were analyzed. Coding sequences (CDS) of *EBNA2*, *EBNA3A*, *EBNA3B*, and *EBNA3C* were extracted using the standard CDS annotations of NC_007605.1 and NC_009334.1 as references, and principal component analysis (PCA) was performed on codon RSCU values. The scores of PC1 and PC2 are shown. Point colors indicate different EBV types. Type 1 and type 2 EBV are clearly separated along the PC1 axis, and sequences with unknown typing were excluded from subsequent typing-related analyses.

To assess the genetic differentiation between NPC-EBV and GC-EBV, a genome-wide association study (GWAS) was conducted based on 205 complete EBV genomes (see Methods for details), and SNP effect sizes (*β*) and odds ratios (*OR* = *exp*(*β*)) were calculated to quantify their association with cancer origin [7,15,16]. The GWAS identified 40 SNPs significantly associated with cancer origin (*p* < 2.09 × 10^−5^) across the EBV genome. Several representative SNPs with the strongest association signals or potential functional relevance are highlighted (Figure 1A,B; Supplementary Table S2). Among these, a synonymous C>T substitution exhibited the strongest association signal (*β* = 0.3195, SE = 0.0387, *p* = 1.80 × 10^−14^, AF = 2.9%). Carriage of the effect allele at this locus significantly increased the odds of an EBV genome being classified as GC-EBV, corresponding to an odds ratio of approximately 1.38.

In addition to this primary locus, several low-frequency SNPs (AF 1.5–2.4%) with consistent effect directions were identified as GC-EBV–associated variants across multiple genomic loci, predominantly distributed within coding regions such as the *EBNA*, *BALF* and *LMP* gene families. Among these, 25 loci are located within coding regions and included both synonymous and nonsynonymous changes. Nonsynonymous and stop-gain variants may alter protein structure or function, with some potentially affecting key functional domains, suggesting functional relevance in EBV-associated carcinogenesis. Notably, a cluster of tightly linked SNPs formed a protective haplotype block within these coding regions (*β* = −0.3721, SE = 0.0568, *p* = 4.65 × 10^−10^, AF = 5.9%), corresponding to an odds ratio of approximately 0.69.

Additional other genome-wide significant variants were identified within these gene regions, exhibiting moderate risk or protective effects. Overall, low-frequency variants (AF < 5%) primarily confer increased GC-EBV risk (*β* > 0), whereas protective variants exhibit intermediate to high allele frequencies (5.4–22.9%), indicating a genetic architecture in the EBV genome where rare, high-effect risk variants coexist with moderate- to high-frequency protective variants.

To comprehensively characterize genome-wide differences between NPC-EBV and GC-EBV, we performed principal component analysis (PCA) based on genome-wide SNPs (Figure 1C). The results showed a partially separated distribution of NPC-EBV and GC-EBV along the first principal component (PC1) axis, suggesting systematic differences in the overall genomic architecture of EBV associated with distinct tumor types. In addition, PCA based on genome-wide SNPs showed that the type 2 EBV sequence clustered within the distribution of type 1 EBV sequences in the PCA space (Figure S1A).

To explore the potential specificity between EBV and different cancer types, we collected and incorporated whole-genome EBV sequences from non-cancer sources for phylogenetic analysis, and conducted stratified analyses by EBV subtype, sampling status, and geographic origin. The results showed that, at the level of EBV typing, there was clear genomic divergence between EBV type 1 and type 2. Across different geographic backgrounds, EBV sequences derived from non-cancer sources did not form an independent cluster but were instead dispersed and interspersed among the NPC- and GC-associated EBV branches. Meanwhile, EBV sequences associated with NPC and GC were distributed across multiple lineages in the phylogenetic tree and overall exhibited a certain degree of clustering separation (Figure S1B).

Overall, among SNPs reaching genome-wide significance, low-frequency variants (AF < 5%) are primarily associated with increased risk (*β* > 0), whereas protective variants exhibit higher allele frequencies (5.4–22.9%), indicating a genetic architecture in the EBV genome where rare, high-effect risk variants coexist with moderate- to high-frequency protective variants.

### 2.2. Nucleotide Composition and Third-Position Base Preference of EBV Core Genes

Given that the GWAS results indicated that cancer type–associated significant SNPs were predominantly enriched within the *EBNA*, *LMP*, and *BALF* gene families, and considering that the oncogenic potential of EBV is largely mediated by a set of latent and lytic functional genes that are extensively involved in host signaling regulation, immune evasion, and cellular transformation, we further conducted a systematic analysis of the evolutionary characteristics of these core gene families at the codon sequence level [14,17,18,19]. To assess the influence of nucleotide composition on codon usage bias, we analyzed the overall base composition of representative genes from the EBNA, LMP, and BALF families.

At the whole-gene nucleotide level, base composition varied among genes but consistently exhibited higher GC content than AT content across all genes. GC content ranged from 51.63% to 66.83%, with *BALF3* (66.83%), *BALF2* (64.86%), *BALF5* (62.30%), and *EBNA1* (63.61%) displaying relatively higher GC abundance, whereas *LMP2B* (51.63%) and *LMP2A* (53.14%) showed comparatively lower GC content. Correspondingly, AT content accounted for 33.17–48.37% across genes and was consistently lower than GC content, indicating an overall GC-rich genomic characteristic of these EBV functional genes. This observation is consistent with the well-recognized GC-rich nature of the EBV genome [20].

Analysis of synonymous codon third-position nucleotide composition revealed a pronounced preference for C3 and G3 across all genes. C3 content ranged from 19.35% to 41.94%, and G3 content ranged from 13.48% to 39.88%, both exceeding the proportions of A3 (11.57–26.34%) and T3 (9.36–33.34%). For example, in *EBNA1*, *BALF2*, and *BALF5*, the combined proportions of C3 and G3 each exceeded 60%. From a functional gene family perspective, lytic cycle–associated genes (*BALF* family) generally exhibited higher GC content and a stronger C3/G3 preference, whereas latent genes (*LMP* family and a subset of *EBNA* genes) showed relatively higher proportions of A3 or T3. Overall, EBV core oncogenic genes were characterized by GC-rich genomes and a predominant C/G bias at the synonymous third codon position.

### 2.3. Codon Usage Patterns of EBV Core Genes Exhibit Type-Associated Structure with Mild Cancer-Related Shifts

Relative synonymous codon usage (RSCU) analysis is commonly employed to characterize synonymous codon usage preferences and potential selective pressures across genes or lineages [9]. In this study, RSCU values were calculated for 13 key EBV genes and subjected to principal component analysis (PCA). The first two principal components (PC1 and PC2) accounted for the majority of variation in synonymous codon usage and were therefore used to assess overall divergence patterns among genes at the codon usage level.

PCA revealed distinct separation patterns for a subset of EBV genes in RSCU space (Figure 1D). Notably, EBNA2 displayed a clear clustering pattern according to EBV type, indicating that its synonymous codon usage characteristics are highly associated with viral typing. Similar type-dependent clustering trends were also observed for EBNA3A, EBNA3B, and EBNA3C, suggesting that these genes may serve as auxiliary markers for EBV typing (Figure 1D and Figure S2A). These results are highly consistent with established EBV classification schemes based on sequence features [8,21]. The RSCU analysis yielded a classification framework consistent with the established EBV typing system. Based on this framework, EBV sequences from public databases without explicit type annotations were assigned to type 1 EBV (I-EBV) or type 2 EBV (II-EBV), while sequences that could not be robustly clustered were categorized as unknown EBV (Unknown-EBV). PCA analysis of *EBNA2* based on sequence SNPs revealed clear separation patterns, which were consistent with the classification results of this gene based on RSCU. Meanwhile, in the PCA analysis of *EBNA3* based on sequence SNPs, type 1 and type 2 also showed a certain degree of distinction (Figure S1B). Unknown-EBV sequences were retained in analyses not requiring explicit EBV typing, whereas they were excluded from type-stratified analyses to avoid potential misclassification bias.

**Figure 2:**
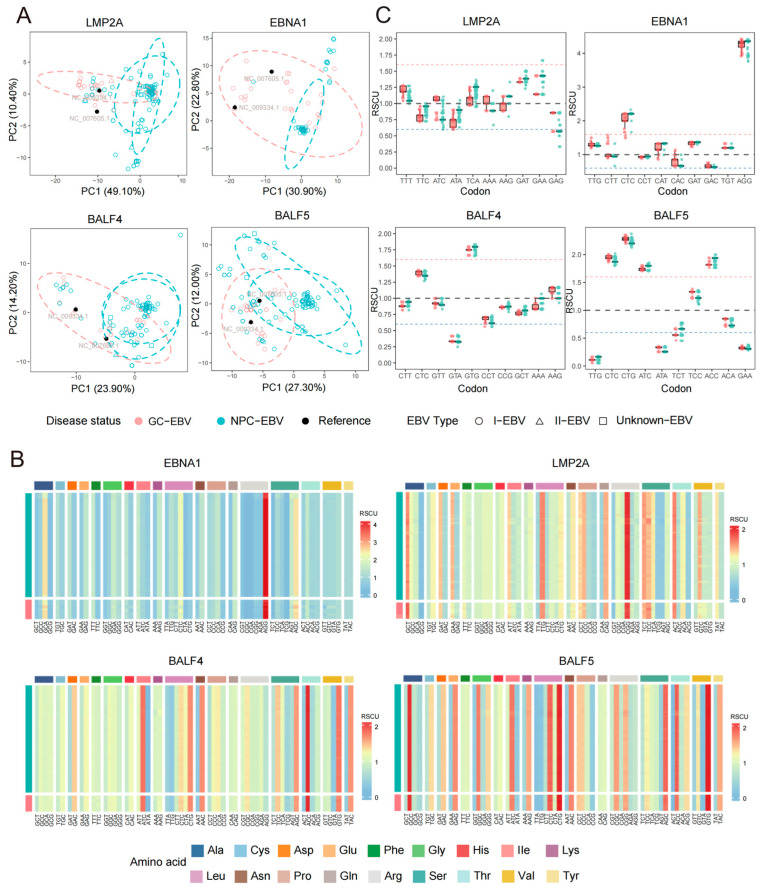
Codon usage bias analysis based on relative synonymous codon usage (RSCU) values of 59 synonymous codons. (**A**) PCA based on RSCU values of 59 synonymous codons for *LMP2A*, *EBNA1*, *BALF4*, and *BALF5* genes. The first two principal components (PC1 and PC2) are shown. Points are colored according to cancer background and shaped according to EBV type. Ellipses indicate 95% confidence intervals and are used to assess clustering and separation patterns among groups. (**B**) Hierarchical clustering heatmap of RSCU values for the 59 synonymous codons in *LMP2A*, *EBNA1*, *BALF4*, and *BALF5*. The top annotation bar indicates the corresponding amino acid categories for each codon, and the left annotation bar denotes cancer background. The color gradient reflects relative RSCU values, illustrating differences in codon usage preferences between cancer backgrounds. (**C**) Boxplots comparing the top 10 synonymous codons with the largest variation in RSCU values within each gene, identified using the Kruskal–Wallis nonparametric test. Box colors represent GC-EBV and NPC-EBV. Red, blue, and gray dashed lines indicate RSCU values of 1.6, 0.6, and 1, corresponding to overrepresented, underrepresented, and unbiased codon usage, respectively.

Building on this framework, we further compared RSCU patterns by jointly considering cancer origin (NPC vs. GC) and EBV type. The results showed that the RSCU patterns of *LMP2A* and *EBNA1* segregated clearly into two distinct groups across different tumor backgrounds, indicating a higher sensitivity to tumor origin. Genes from the *BALF* family exhibited overall stability in RSCU patterns, although moderate differences were still observed, mainly involving *BALF4* and *BALF5*, and the influence of EBV type on these genes was comparatively limited (Figure 2A and Figure S2B).

Among *EBNA1*, *LMP2A*, *BALF4*, and *BALF5*, synonymous codon usage differences were particularly pronounced. Overrepresented codons with median RSCU values greater than 1.6 were predominantly GC-ending codons (*EBNA1*: 3/5; *LMP2A*: 1/3; *BALF4*: 7/7; *BALF5*: 11/11), such as CTC and AGG in *EBNA1*; ATC, GTG, and ACC in *BALF4*; and CTC, CTG, and ATC in *BALF5*. Meanwhile, underrepresented codons with median RSCU values below 0.6 were mainly AT-ending codons (*EBNA1*: 9/15; *LMP2A*: 4/8; *BALF4*: 8/10; *BALF5*: 14/17). Notably, these distribution patterns were highly consistent between NPC and GC backgrounds (Figure 2B).

To further evaluate differences in codon usage preferences between NPC- and GC-associated EBV genomes, RSCU values for each gene were compared using the Kruskal–Wallis test with cancer origin as the grouping variable. For each gene, the ten synonymous codons exhibiting the largest differences were selected for detailed comparison. *LMP2A* and *EBNA1* showed marked tumor background-dependent differences in RSCU preferences, while *BALF4* and *BALF5* displayed largely conserved codon usage patterns with only minor but consistent variations. Specifically, in *LMP2A*, most synonymous codons exhibited overall upward or downward shifts in RSCU distributions between NPC- and GC-associated sequences. For example, the median RSCU value of ATC exceeded 1 in GC-EBV sequences but was close to or slightly below 1 in NPC-EBV sequences. In *EBNA1*, the differences were more concentrated and of greater magnitude: CTC and AGG consistently showed median RSCU values greater than 1.6 in both NPC- and GC-associated sequences, representing stably overrepresented codons, with further elevation of median values observed in NPC-EBV. In contrast, the median RSCU value of CAC was close to 1 in GC-EBV but approached 0.6 in NPC-EBV.

By comparison, *BALF* family genes exhibited more compact RSCU distributions between NPC-EBV and GC-EBV, with only a limited number of codons showing mild but consistent cancer-related shifts. For instance, in BALF4, the median RSCU value of AAG was higher in GC-EBV than in NPC-EBV, whereas GTG was overrepresented in both cancer backgrounds but showed a slightly higher median value in NPC-EBV. In BALF5, the codons CTC, CTG, ATC, TCC, and ACC consistently exhibited strong usage preferences, with median RSCU values significantly greater than 1. Among these, the median RSCU values of CTC, CTG, and TCC were higher in GC-associated EBV than in NPC-associated EBV, indicating a mild tendency toward tumor background-associated shifts in codon usage preference (Figure 2C). To assess the potential functional relevance of codon usage differences in a cell type-specific context, publicly available tRNA expression data were retrieved from the tsRinCancer database [22]. Data corresponding to stomach adenocarcinoma (STAD) and head and neck squamous cell carcinoma (HNSC) were extracted, with HNSC used as an approximate representation of nasopharyngeal carcinoma. Based on the RSCU analysis, ten codons showing relatively large differences in LMP2A and EBNA1 were selected, and the expression changes of their corresponding tRNA isoacceptors were examined. At the level of numerical distribution, several codons (e.g., ATC and GAG) showed differences in the median log_2_ fold change (log_2_FC) between the two cancer contexts. For example, in LMP2A, the median log_2_FC of the tRNA corresponding to ATC was higher in STAD than in HNSC, consistent with the RSCU analysis in which ATC exhibited a higher median RSCU value in the gastric cancer context. However, given that tRNA expression may be influenced by factors such as tissue origin, tumor heterogeneity, and host genetic background, and in the absence of statistically significant and consistent evidence, the current results remain insufficient to support further functional inference (Figure S3A).

### 2.4. Mutational Pressure and Natural Selection Jointly Shape Codon Usage Patterns of EBV Core Genes

**Figure 3:**
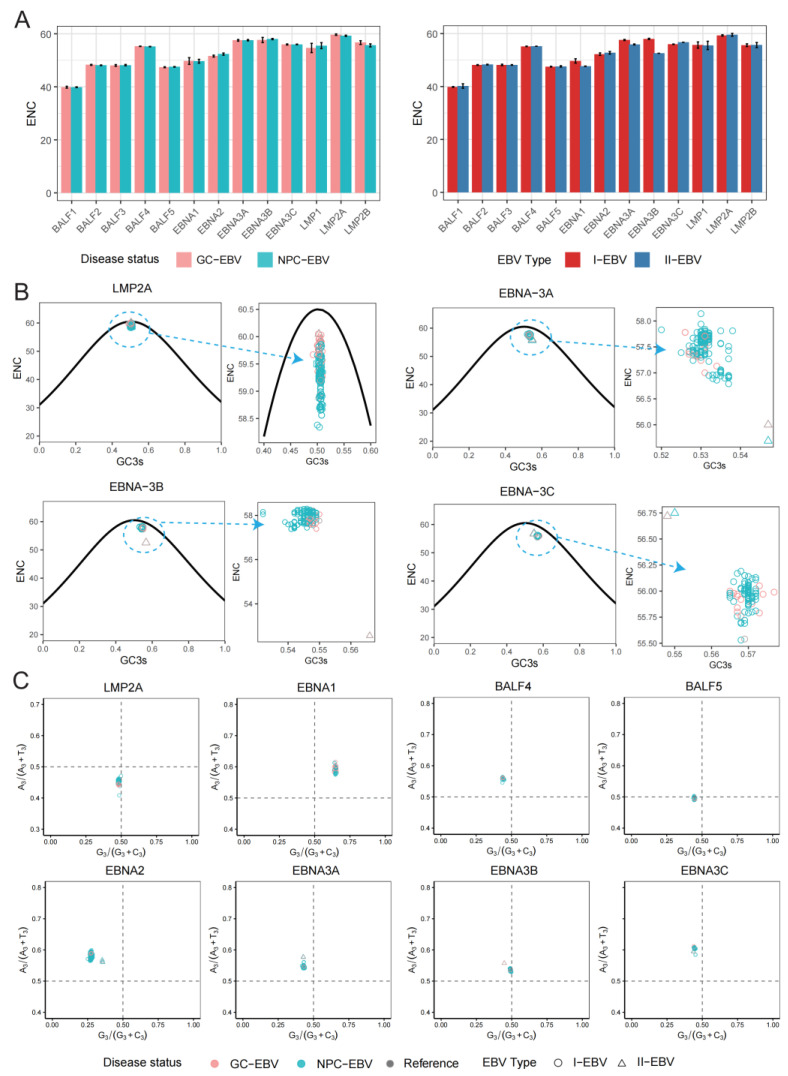
Mechanistic analysis of codon usage bias based on ENC, ENC–GC3s, and PR2 analyses. (**A**) Comparison of mean effective number of codons (ENC) values for 13 EBV genes under different cancer backgrounds and EBV types. Colors indicate different groups. Error bars represent within-group variation (mean ± standard deviation). (**B**) ENC–GC3s plots for *LMP2A*, *EBNA3A*, *EBNA3B*, and *EBNA3C* genes. The solid curve represents the theoretical expected ENC values under mutation pressure alone. Points are colored according to cancer background and shaped according to EBV type. The region highlighted by the dashed orange circle on the right is shown as a magnified view, with arrows indicating the corresponding positions. (**C**) Parity Rule 2 (PR2) analysis of *LMP2A*, *EBNA1*, *EBNA2*, *EBNA3*, *BALF4*, and *BALF5* genes to evaluate A/T and G/C usage bias at the third codon position. Points are colored according to cancer background and shaped according to EBV type. The central point (0.5, 0.5) represents the theoretical equilibrium state in the absence of mutation bias or selection pressure.

To quantitatively evaluate the strength of codon usage bias in EBV core oncogenic genes across different cancer backgrounds and viral types, the effective number of codons (ENC) was calculated for each of the 13 target genes [23,24]. Under both NPC and GC backgrounds, ENC values generally fell within the medium-to-high range, spanning 39.85–59.68 in GC-associated sequences and 39.85–59.25 in NPC-associated sequences (Figure 3A). Among these, *BALF1* consistently exhibited the lowest ENC values in both cancer backgrounds (GC: 39.86 ± 0.35; NPC: 39.85 ± 0.14), indicating relatively strong synonymous codon usage bias. In contrast, *LMP2A* showed the highest ENC values in both GC and NPC (GC: 59.68 ± 0.27; NPC: 59.25 ± 0.22), suggesting codon usage patterns approaching random expectation. The remaining genes largely displayed ENC values between 47 and 58, reflecting overall weak codon usage bias.

Across EBV types, ENC values were similarly highly consistent, with an overall range of 39.84–59.54. Although EBNA3A and EBNA3B exhibited ENC differences that were dependent on viral type background, the magnitude of these differences was generally less than 1.0 for most genes, indicating no substantial enhancement of synonymous codon usage bias (Figure 3A).

To further assess the contribution of mutational pressure to EBV synonymous codon usage bias, the relationship between ENC and GC content at the synonymous third codon position (GC3s) was examined by constructing ENC–GC3s plots [25] (Figure 3B and Figure S4A). All observed data points were systematically distributed below Wright’s theoretical expectation curve and formed relatively stable clusters beneath the curve, indicating that codon usage patterns of EBV core genes cannot be explained by mutational pressure alone and that natural selection also plays a substantial role.

Notably, distinct distribution patterns were observed among genes in ENC–GC3s space. *LMP2A* consistently exhibited higher ENC values, with GC-associated sequences approaching ENC ≈ 60, further supporting its near-random codon usage pattern. EBNA2 and members of the *EBNA3* family generally displayed GC3s values greater than 0.5, indicating a pronounced GC bias at the third codon position. Moreover, *EBNA3A*, *EBNA3B*, and *EBNA3C* showed clearer separation along the GC3s axis between EBV types. Specifically, *EBNA3A* and *EBNA3B* exhibited higher GC3s and lower ENC values in II-EBV compared with I-EBV, whereas *EBNA3C* displayed the opposite pattern (Figure 3B), suggesting that even within the same gene family, individual members may be subject to distinct evolutionary constraints.

Given that mutational pressure and natural selection are the two principal forces shaping codon usage bias, Parity Rule 2 (PR2) analysis was further applied to evaluate base usage balance at the synonymous third codon position (Figure 3C and Figure S4B) [26]. LMP family genes were predominantly distributed in regions with A3/(A3 + T3) < 0.5 and G3/(G3 + C3) < 0.5, indicating a preference for T and C at the third codon position. In contrast, *EBNA* family genes exhibited more heterogeneous distribution patterns. *EBNA1* showed stable enrichment in regions with G3/(G3 + C3) > 0.5, whereas *EBNA2* and *EBNA3* family members tended toward G3/(G3 + C3) < 0.5, indicating marked divergence in third-position GC preference among *EBNA* genes. Meanwhile, EBV typing exerted a modulatory effect on base usage frequencies, reflecting type-associated constraints on nucleotide composition during viral evolution.

For lytic cycle-associated genes, BALF4 and BALF5 were primarily distributed in regions with G3/(G3 + C3) < 0.5. BALF4 showed a preference for A over T at the third codon position, whereas BALF5 exhibited a more balanced usage of A and T, suggesting subtle gene-specific differences in third-position codon selection among lytic genes.

**Figure 4:**
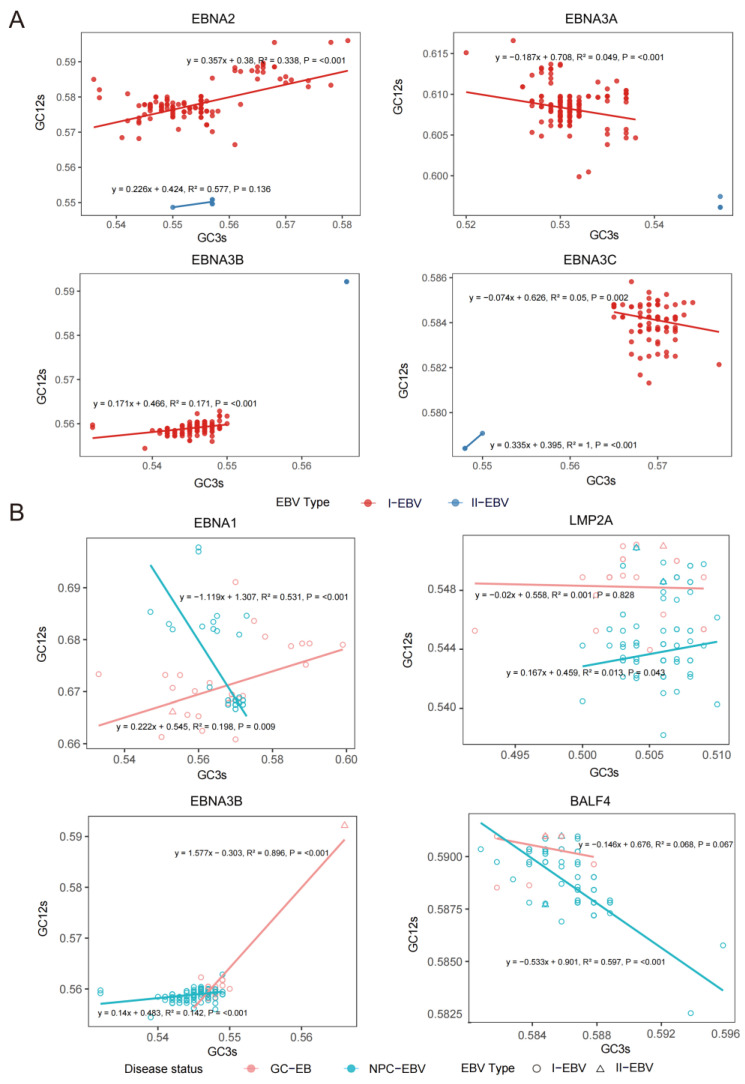
Neutrality plot analysis of EBV gene codon usage under different grouping backgrounds. (**A**) Neutrality plot analysis of *EBNA2* and *EBNA3* genes under different EBV typing backgrounds. Points and solid lines are colored according to EBV type. (**B**) Neutrality plot analysis of *EBNA1*, *LMP2A*, *EBNA3B*, and *BALF4* genes under different cancer backgrounds. Points and solid lines are colored according to cancer background, with point shapes indicating EBV type. In all neutrality plots, GC12s (mean GC content at the first and second codon positions) is plotted on the *y*-axis, and GC3s (GC content at the third codon position) on the *x*-axis. Solid lines indicate linear regression between GC12s and GC3s. Regression equations, coefficients of determination (R^2^), and *p* values are shown to assess the relative contributions of mutation pressure and natural selection to codon usage bias.

Neutrality plot analysis further quantified the relative contributions of mutational pressure and natural selection in shaping codon usage patterns (Figure 4A and Figure S5A) [25,27]. When stratified by EBV type, *EBNA2* exhibited weak positive regression slopes in both type 1 and type 2 EBV, indicating limited coupling between GC3 and GC12. This suggests that mutational pressure alone is insufficient to explain its codon usage pattern. Within the *EBNA3* family, correlations were generally weak, and negative slopes were observed in certain contexts (e.g., *EBNA3A* and *EBNA3C*), indicating inverse variation between synonymous and nonsynonymous sites. Such negative correlations are inconsistent with a purely mutation-driven model and more likely reflect functional constraints on amino acid composition and the action of purifying selection. For *EBNA3C*, the positive slope was relatively higher in type 2 EBV than in type 1 EBV, suggesting comparatively stronger mutational influence in type 2 strains, although selective forces still appear to play an important role overall.

Neutrality analysis based on cancer background revealed significant differences in the evolutionary forces acting on core genes between NPC-EBV and GC-EBV. Specifically, In *EBNA1*, NPC-EBV displayed a negative slope, whereas GC-EBV showed a positive slope below 0.5. The negative correlation in the NPC background indicates opposing evolutionary trends between GC3 and GC12, consistent with stronger selective constraints. In contrast, although a positive association was observed in the GC background, the weak coupling suggests that while mutational pressure may contribute to codon variation, selection likely remains predominant. For BALF4, negative slopes were observed in both cancer backgrounds, indicating a consistent inverse relationship between GC3 and GC12 (Figure 4B and Figure S5B).

Taken together, results from ENC analysis, ENC–GC3s plots, PR2 bias assessment, and neutrality analysis indicate that selective pressures acting on individual EBV genes differ markedly in a cancer background-dependent and/or viral type-dependent manner. In particular, *EBNA1* and *LMP2A* appear to be subject to distinct evolutionary forces under NPC versus GC conditions. Moreover, in GC-associated EBV sequences, the effect of mutational pressure shows a strong association with viral type, suggesting an interaction between viral lineage and tumor-specific evolutionary constraints. Overall, these findings suggest that the evolutionary dynamics of EBV genes are not governed by a single mechanism but rather reflect the combined effects of mutational pressure and selective constraint. In particular, inverse GC3–GC12 relationships are more consistent with gene-specific selective constraints than with mutation-driven processes alone.

### 2.5. Dinucleotide Composition Bias and Its Influence on Codon Usage Patterns

In addition to mutational pressure and natural selection, dinucleotide composition bias has also been recognized as an important factor influencing synonymous codon usage bias [28]. To systematically evaluate dinucleotide composition features and their potential evolutionary implications in EBV core genes, the relative abundances (observed/expected, O/E) of all 16 dinucleotides were calculated for each analyzed gene.

**Figure 5:**
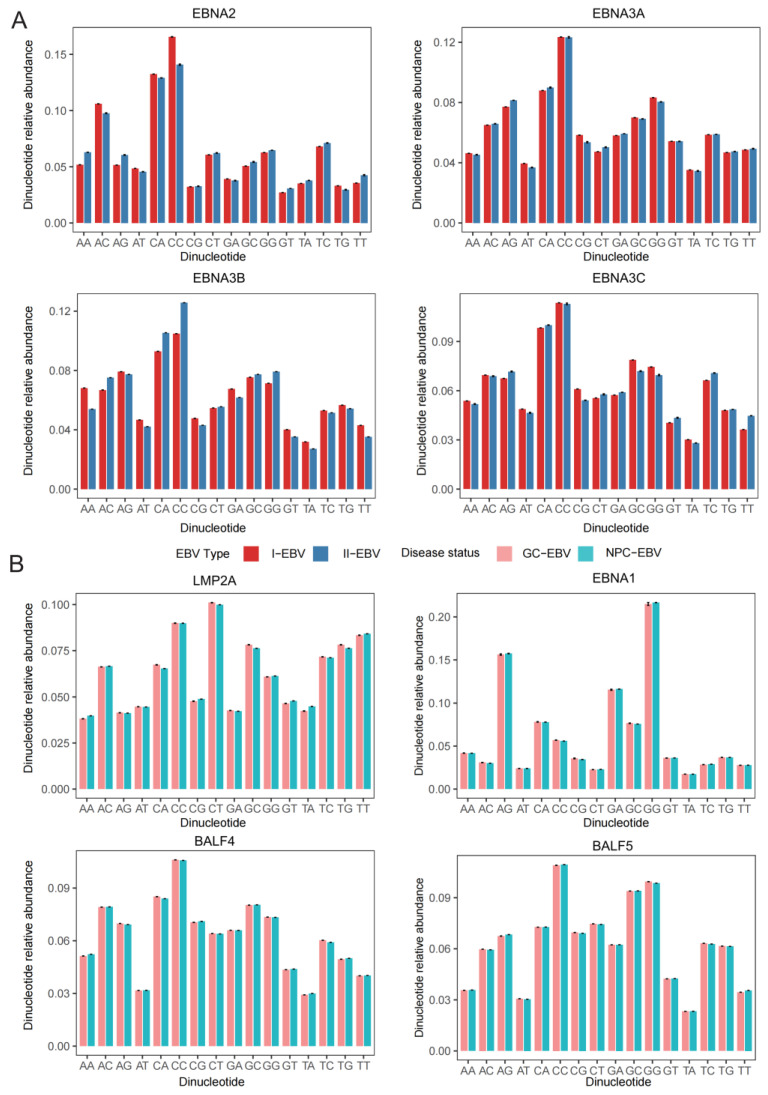
Dinucleotide relative abundance analysis of EBV gene codon usage under different grouping backgrounds. (**A**) Relative abundances of 16 dinucleotides in *EBNA2* and *EBNA3* genes under different EBV typing backgrounds. Bar colors represent EBV types. (**B**) Relative abundances of 16 dinucleotides in *LMP2A*, *EBNA1*, *BALF4*, and *BALF5* genes under different cancer backgrounds. Bar colors represent cancer background. In both analyses, error bars indicate within-group variation (mean ± standard deviation), reflecting variability in dinucleotide usage preferences across groups.

The results showed that dinucleotide usage patterns in all samples deviated significantly from random expectations, as indicated by highly significant chi-square test results (χ^2^, *p* < 0.001), suggesting pervasive non-random dinucleotide usage across the EBV genome (Figure 5A and Figure S6A). Among all analyzed genes, CpG and TpA dinucleotides were consistently and markedly underrepresented, with O/E values generally below 1 and highly concordant suppression patterns observed across genes. CpG suppression was particularly pronounced, consistent with the widely reported CpG depletion characteristic of viral genomes. Concomitant with CpG suppression, certain dinucleotides, such as CpT and TpG, exhibited varying degrees of relative enrichment across multiple genes.

When examined from a functional phase perspective, lytic cycle–associated genes (*BALF* family) and latent phase-associated genes (*EBNA* and *LMP* families) displayed discernible differences in dinucleotide usage preferences. Overall, latent genes showed more pronounced CpG suppression, whereas this trend was relatively attenuated in *BALF* family genes.

Further comparisons across EBV types revealed notable differences in dinucleotide composition for *EBNA2* and *EBNA3* genes. Specifically, in EBNA2, relative abundances of nearly all dinucleotides differed between EBV type 1 and type 2, with the most pronounced difference observed for CpC. In *EBNA3*, dinucleotides including ApT, CpA, CpC, GpC, and GpT also exhibited varying degrees of type-associated differences. Together, these patterns suggest that distinct EBV types may have undergone gene-specific sequence divergence, potentially reflecting differences in their evolutionary histories (Figure 5A and Figure S6A).

When stratified by tumor type (NPC vs. GC), dinucleotide composition differences were generally modest for most analyzed genes, particularly among BALF family genes, which exhibited high compositional stability across cancer backgrounds. However, certain latent genes still showed detectable cancer-associated differences. For example, in LMP2A, relative abundances of CpA, CpT, GpC, and TpG differed between NPC- and GC-associated samples. In EBNA1, the relative abundance of GpG also varied to some extent, suggesting that dinucleotide composition of latent genes may be influenced, at least in part, by differences in tumor microenvironments or host backgrounds (Figure 5B and Figure S6B).

Overall, dinucleotide composition bias in EBV core genes is highly non-random and exhibits gene-specific, type-associated, and cancer-related heterogeneity. These patterns suggest that dinucleotide preferences may contribute to shaping EBV codon usage bias by modulating synonymous codon availability and translation-related constraints.

## 3. Discussion

Epstein–Barr virus (EBV) is a well-established etiological agent of multiple lymphoid and epithelial malignancies and is characterized by two major, long-standing viral types [8,13]. In this study, we systematically investigated EBV genetic differentiation and evolutionary features across two epithelial malignancies in which EBV is most prevalent—NPC and GC—as well as across EBV types, by integrating genome-wide sequence variation, synonymous codon usage bias, and dinucleotide composition analyses. By integrating population-level SNP differentiation with coding-level synonymous codon usage patterns, we present a multi-scale descriptive framework to characterize structural differences in the EBV genome across distinct tumor-associated contexts.

Genome-wide SNP-based PCA and GWAS analyses revealed a clear but incomplete separation between EBV genomes derived from NPC and GC, indicating measurable genetic differentiation associated with tumor background. Multiple SNPs reached genome-wide significance, encompassing both low-frequency, high-effect GC-associated risk variants and relatively common protective haplotypic blocks. The coexistence of these “rare high-effect variants” and “common protective variants” suggests a complex population structure of EBV across different tumor-associated contexts; however, these patterns may also be partly attributable to underlying viral lineage structure or geographic stratification.

Despite cancer-associated differentiation at the SNP level, synonymous codon usage patterns exhibited pronounced gene-specific heterogeneity. PCA based on relative synonymous codon usage (RSCU) demonstrated that *EBNA2* and *EBNA3* family members clustered strongly according to EBV type, with clustering patterns highly consistent with established EBV typing schemes. This result suggests that the codon usage preferences of these genes are largely associated with stable viral lineage structure rather than being directly linked to tumor background, reflecting their core functional roles in EBV biology.

In contrast, *EBNA1* and *LMP2A* displayed greater sensitivity to tumor background, showing systematic yet moderate shifts in codon usage patterns between NPC- and GC-associated sequences. These differences are primarily manifested as changes in the usage frequencies of a limited subset of over- or under-represented codons rather than a global restructuring of the codon usage profile, suggesting a potential association between codon usage patterns and tumor background. However, it should be noted that such differences may also be influenced by phylogenetic background, viral lineage composition, or geographic origin, and the current study design does not allow these factors to be fully disentangled.

ENC–GC3s analyses consistently placed all genes below the expected curve, indicating that mutation pressure alone cannot account for EBV codon usage patterns and that natural selection plays a substantial role. PR2 bias analyses further revealed pronounced differences in third-codon-position nucleotide preferences among gene families, with *EBNA*, *LMP*, and *BALF* genes following distinct compositional trajectories. Neutrality plot analyses quantitatively delineated the relative contributions of mutational pressure and natural selection across different genes and viral lineages; however, these results primarily reflect statistical associations rather than providing direct evidence for specific selective mechanisms.

Notably, although lytic cycle-associated *BALF* genes harbored several cancer-associated SNPs at the genome-wide level, their synonymous codon usage patterns remained highly stable across tumor backgrounds and EBV types. RSCU distributions, ENC values, and dinucleotide relative abundances in *BALF4* and *BALF5* showed only minimal, directionally consistent shifts. This apparent decoupling—cancer-associated differentiation at the SNP level coupled with stability at the codon usage level—suggests that EBV is subject to distinct selective regimes at different evolutionary scales. While individual nucleotide substitutions may accumulate without substantially altering overall coding architecture, higher-order features such as codon usage bias in *BALF* genes appear to be constrained by strong functional conservation [17,18].

From a biological perspective, *BALF* family genes are primarily involved in lytic replication and virion assembly, processes that require rapid and accurate protein expression within a limited temporal window. Sustained selective pressure on translational efficiency, fidelity, and compatibility with the host translational machinery may therefore enforce long-term stability in their synonymous codon usage patterns across diverse genetic and tumor-associated backgrounds [29,30]. By contrast, genes primarily expressed during the latent infection phase, such as members of the *EBNA* and *LMP* families, function in a context of continuous but tightly regulated expression. Unlike lytic genes, which rely on high-level, efficient protein production, latent genes may be subject to different selective pressures associated with long-term persistence and regulatory control. Although this study did not directly measure translation efficiency, relatively high variability in synonymous codon usage was observed among latent genes [8,31]. Further, although this study systematically reveals associations between codon usage preferences of certain EBV genes and tumor background, the inherently observational nature of evolutionary analyses precludes the establishment of a direct causal relationship between EBV evolutionary trajectories and tumorigenesis.

In summary, our findings demonstrate that synonymous codon usage patterns in EBV are jointly shaped by natural selection, mutation pressure, and dinucleotide composition under different epithelial cancer contexts (NPC versus GC) and EBV typing backgrounds. Codon usage in *EBNA2* and *EBNA3* is predominantly constrained by EBV type–associated evolutionary forces, whereas *EBNA1* and *LMP2A* exhibit cancer-associated modulation driven by varying contributions of selection and mutation pressure. In contrast, lytic cycle–associated *BALF* genes are subject to stronger functional conservation, maintaining highly stable codon usage patterns across genetic backgrounds. Together, this multi-layered evolutionary analysis advances our understanding of EBV type differentiation and adaptive evolution in epithelial malignancies and provides new insights relevant to EBV classification and tumor-associated viral evolution.

## 4. Method

### 4.1. Source of Target Sequences

All available EBV complete genome sequences were retrieved from the NCBI database using the query “txid10376[organism:exp] AND biomol_genomic[prop]”. The downloaded genome sequences were compiled to construct a local EBV genome database for subsequent analyses. Based on the GenBank annotation files of reference genomes, coding sequences (CDSs) of target genes were extracted from two reference EBV genomes (NC_007605.1; NC_009334.1) using a custom analysis pipeline and used as query sequences. The CDSs were then aligned against the local EBV genome database using BLASTn (v2.14.1+), with the output format set to -outfmt 6. For each gene in each genome, only the alignment with the highest bitscore was retained to ensure the extracted sequence represented the best homologous match. Coordinates of the selected alignments were used to extract the corresponding CDS regions, and redundant sequences were removed to generate a non-redundant CDS dataset for each gene across all genomes. Only sequences derived from nasopharyngeal carcinoma (NPC) and gastric cancer (GC) samples were retained for downstream analyses. In total, CDSs of 13 functionally important EBV genes were extracted, including *LMP1*, *LMP2A*, *LMP2B*, *EBNA1*, *EBNA2*, *EBNA3A*, *EBNA3B*, *EBNA3C*, *BALF1*, *BALF2*, *BALF3*, *BALF4*, and *BALF5*. These CDS sequences were subsequently used for codon-level analyses. Multiple sequence alignments for phylogenetic analyses were performed using MAFFT (v7.526) [32] with default automatic parameters (mafft --auto). The EBV whole-genome sequences not associated with infection were obtained from EBV whole-genome sequences derived from healthy individuals that had been previously submitted in published studies [7].

### 4.2. Genome-Wide SNP Identification and Population Genetic Analyses

Complete EBV genome sequences with clearly annotated cancer origin (nasopharyngeal carcinoma, NPC; gastric cancer, GC) were retrieved from the NCBI database. All genomes were aligned to the EBV reference strain NC_007605.1 to ensure a unified coordinate system for variant calling. Whole-genome alignments were performed using minimap2 (v2.28-r1209) with the asm5 preset [33], followed by sorting and indexing with samtools (v1.21) [34]. Single nucleotide polymorphisms (SNPs) were identified using bcftools mpileup and bcftools call, explicitly specifying a haploid genome model. Only SNPs were retained, and insertion–deletion variants were excluded. Variant statistics were assessed using bcftools stats [35]. Variant statistics were generated using bcftools stats. Individual sample VCF files were merged with bcftools merge (--missing-to-ref), retaining only biallelic SNPs for downstream analyses. Only biallelic SNPs were retained for downstream analyses. The merged VCF was converted to PLINK binary format using PLINK (v1.9) [36], allowing non-standard chromosome names.

To account for genetic relatedness and population structure among EBV genomes, a genomic relationship matrix (GRM) was constructed using GEMMA 0.98.3 with the centered relatedness matrix option [37]. This approach estimates pairwise genetic similarity among viral genomes based on genome-wide SNP data. Genome-wide association analysis between NPC-derived and GC-derived EBV genomes was performed using a univariate linear mixed model (LMM) implemented in GEMMA; the LMM incorporates the GRM as a random effect to control for cryptic population structure and genetic background effects. Disease status (GC vs. NPC) was treated as a binary phenotype provided in the phenotype file [37].

Prior to GWAS, the merged biallelic SNP dataset (VCF format) was converted to GDS format using the snpgdsVCF2GDS function, retaining only biallelic SNPs to facilitate efficient computation. SNPs were subsequently pruned for linkage disequilibrium (LD) using snpgdsLDpruning (R^2^ threshold = 0.6, sliding window = 1 kb), and SNPs with low minor allele frequency (MAF < 0.05) or located on non-autosomal chromosomes were excluded to reduce redundancy and potential bias. The resulting dataset was used for principal component analysis (PCA). In the GEMMA-based LMM GWAS, SNP effects on the binary phenotype (GC vs. NPC) were represented by regression coefficients (*β*). For binary traits, *β* can be approximately converted to odds ratios (*OR*s), allowing continuous effect estimates to be interpreted as disease risk ratios consistent with conventional case–control studies:

*OR* ≈ *exp* (*β*)

Wald test statistics were used to evaluate SNP-level associations. Only SNPs with valid *p* values (0 < *p* ≤ 1) were retained for downstream analyses. To correct for multiple testing, both Bonferroni correction and false discovery rate (FDR, Benjamini–Hochberg method) were applied [38]. The Bonferroni-adjusted significance threshold was defined as:

α*Bonf* = 0.05/N

where N represents the total number of SNPs tested.

Genomic inflation was assessed by calculating the genomic inflation factor (λGC) based on the median of the observed chi-square statistics. Population structure and cancer-associated genetic differentiation were assessed using PCA and genome-wide association analysis.

### 4.3. Principal Component Analysis (PCA)

Principal component analysis (PCA) is a multivariate statistical method used to reduce data dimensionality while retaining the major sources of variation. In this study, PCA was applied to both genome-wide SNP data and synonymous codon usage profiles to capture the primary patterns of genetic and compositional variation among EBV sequences. The first two principal components (PC1 and PC2), which explained the largest proportions of total variance, were extracted and visualized to assess clustering patterns and differentiation among groups.

### 4.4. Nucleotide Composition Analysis

After removing stop codons (TAA, TGA, and TAG) as well as ATG and TGG (because methionine and tryptophan are each encoded by a single codon, ATG and TGG, respectively), the compositional parameters of the 13 genes for which CDSs were calculated. The nucleotide frequencies at the third synonymous codon positions (A3s, G3s, C3s, and T3s) were calculated using CodonW (v1.4.4). The nucleotide frequencies of A, T, G, and C were calculated using the CAIcal [39] server (http://genomes.urv.es/CAIcal/; accessed on 30 December 2025).

### 4.5. Analysis of Relative Synonymous Codon Usage (RSCU)

RSCU refers to the relative probability of using a specific codon usage among synonymous codons encoding the same amino acid [40]. An RSCU value of 1 indicates no bias. Specifically, RSCU > 1 reflects preferential usage, with RSCU ≥ 1.6 commonly considered strong overrepresentation, whereas RSCU ≤ 0.6 is generally interpreted as significant avoidance of a codon. RSCU values for 59 codons (excluding ATG [Met], TGG [Trp], the three stop codons TAA, TGA, and TAG, and ambiguous bases) were calculated using the CAIcal [39] website (http://genomes.urv.es/CAIcal/; accessed on 30 December 2025). For each synonymous codon, the optimal codon was chosen based on its highest number of occurrences and largest RSCU. Synonymous codon usage data for hosts were retrieved from the Codon Usage Database (http://codonstatsdb.unr.edu/; accessed on 30 December 2025) [41]. The RSCU values were calculated using the following formula [42]:

$${RSCU}_{ij}=\frac{X_{ij}}{\sum_{j=1}^{n_{i}}X_{ij}}n_{i}$$

where *X_ij_* represents the number of occurrences of the *j*th codon for the *i*th amino acid, which has *n_i_* types of synonymous codons. Based on the computed RSCU values for five genes, heatmaps were generated using R (v4.4.2) to visually illustrate differences in codon usage patterns among different viral lineages.

### 4.6. Analysis of Dinucleotide Relative Abundance and Characterization

To understand the impact of dinucleotide frequencies on codon usage selection and identify overrepresented dinucleotides, the occurrence frequencies of all 16 possible dinucleotides within coding sequences were calculated. DAMBE (v7.3.7) [43] software was used to compute the relative abundance of dinucleotides. The formula for calculating the dominance ratio of the 16 dinucleotides is as follows:

$$P_{xy}=\;\frac{f_{xy}}{f_{x}f_{y}}$$

where *f*_*x*_, *f*_*y*_, and *f*_*xy*_ denote the occurrence rate of nucleotide *X*, the prevalence of nucleotide *Y*, and the recorded occurrence frequency of the dinucleotide *XY*, respectively. When *P*_*xy*_ exceeds 1.23 (or falls below 0.78), the dinucleotide *XY* is deemed as being overrepresented (or underrepresented).

### 4.7. Analysis of Effective Number of Codons (ENC)

ENC is a method used to describe the strength of preference for the usage of synonymous codons [44]. The ENC value ranges from 20 (when only one codon is used) to 61 (when all synonymous codons are equally used). A lower ENC value indicates a stronger codon preference. An ENC value below 35 suggests a strong codon preference. The ENC value can be calculated using CodonW (v1.4.4). The formula for calculating the ENC value is as follows:

$$ENC=2+\;\frac{9}{{\bar{F}}_{2}}+\frac{1}{{\bar{F}}_{3}}+\frac{5}{{\bar{F}}_{4}}+\frac{3}{{\bar{F}}_{6}}$$

where ${\bar{F}}_{K}(k=2,3,4,6)$ represents the means of the *k*-fold degenerate amino acids, which is calculated as outlined below:

$${\bar{F}}_{k}=\frac{n\sum_{j=1}^{i}{\left(\frac{n_{j}}{n}\right)}^{2}-1}{n-1}$$

where *n* is the total count of occurrences for the codons associated with a particular amino acid; *n*_*j*_ represents the count of occurrences for the specific *j*th codon related to that amino acid.

### 4.8. ENC-GC3s Plot Analysis

ENC-GC3s plots are often used to visualize whether mutation pressure is a major determinant of codon usage bias. An ENC-GC3s plot involves constructing a scatter plot with GC3s as the independent variable and the ENC value as the dependent variable. If mutational pressure is the sole driving factor behind codon usage bias, the points on the plot will lie on a curve that can be predicted when the value of the ENC depends only on genomic composition, and computed as follows:

$${ENC}_{expected}=2+GC3s+(\frac{29}{{\left(GC3s\right)}^{2}+{\left(1-GC3s\right)}^{2}})$$

Alternatively, if the points are below the standard curve, it suggests that factors other than mutational pressure influence codon usage bias. ENC values and GC3s were calculated using CodonW (v1.4.4), and R (v4.4.2) was used for plotting.

### 4.9. The Parity Rule 2 (PR2) Analysis

A PR2 plot is a method of studying the composition of codon bases. CAI website (http://genomes.urv.es/CAIcal/; accessed on 30 December 2025) was used to calculate the A3%, C3%, G3%, and T3% values. The comparison of A3/(A3 + T3) with G3/(G3 + C3) is used to assess the relationship between mutation pressure and natural selection. A = T and G = C (i.e., axis values of 0.5 and 0.5), respectively, indicating a balance between mutation pressure and natural selection.

### 4.10. Neutrality Analysis

Regression curves were computed to assess the impacts of mutational pressure and natural selection on codon usage, using the GC12s (*y*-axis) plotted against the GC3s (*x*-axis) relationship. The stronger the correlation, the closer the slope of the regression line is to 1, indicating that codon usage bias is primarily influenced by mutational pressure. Conversely, as the slope of the regression line gradually decreases (even reaching 0), it suggests an increasing role of natural selection pressure on codon usage bias. Neutrality plots were constructed by R (v4.4.2), and regression lines were calculated.

### 4.11. Statistical Analysis and Data Visualization

All statistical analyses and data visualizations were performed using R (v4.4.2). Figures were generated using core R functions and contributed packages for statistical analysis and graphical visualization. Unless otherwise stated, statistical tests were two-sided, and *p* < 0.05 was considered statistically significant.

Phylogenetic relationships were inferred using IQ-TREE (v3.0.1) [45], with the best-fit substitution model selected automatically by ModelFinder Plus (MFP) option (-m MFP). Node support was assessed with ultrafast bootstrap analysis using 1000 replicates. The resulting phylogenetic trees were subsequently annotated and visualized using ChiPlot [46] for aesthetic refinement and interpretation.

## Data Availability

The original contributions presented in the study are included in the article/Supplementary Material. Further inquiries can be directed to the corresponding author.

## Supplementary Materials

The following supporting information can be downloaded at: https://www.mdpi.com/article/10.3390/v18040425/s1, Figure S1: Principal component analysis of NPC-EBV and GC-EBV under EBV typing backgrounds, together with whole-genome phylogenetic analysis including non-cancer samples. Figure S2: Comparison of tRNA isoacceptor expression corresponding to codons with relatively large RSCU differences in *LMP2A* and *EBNA1* between STAD and HNSC. Figure S3: Codon usage bias analysis based on RSCU values of 59 synonymous codons in different genes. Figure S4: Mechanistic analysis of codon usage bias based on ENC–GC3s and PR2 analyses. Figure S5: Neutrality plot analysis of codon usage in different genes under different backgrounds. Figure S6: Dinucleotide relative abundance analysis of EBV gene codon usage under different grouping backgrounds. Table S1: Comprehensive Metadata of EBV Whole-Genome Sequences Used in This Study; Table S2: Association Analysis of EBV Genetic Variants with Functional Annotation.
