# Supplementary material for: Codon Usage Bias Variation and Evolutionary Signatures of Epstein–Barr Virus in Distinct Epithelial Cancers
Source: Viruses. 2026 Mar 31;18(4):425. doi: 10.3390/v18040425 (PMC13119708; doi:10.3390/v18040425)
Supplement: Supplementary file 1 [file viruses-18-00425-s001.zip › viruses-4172337-supplementary_Figures.pdf]

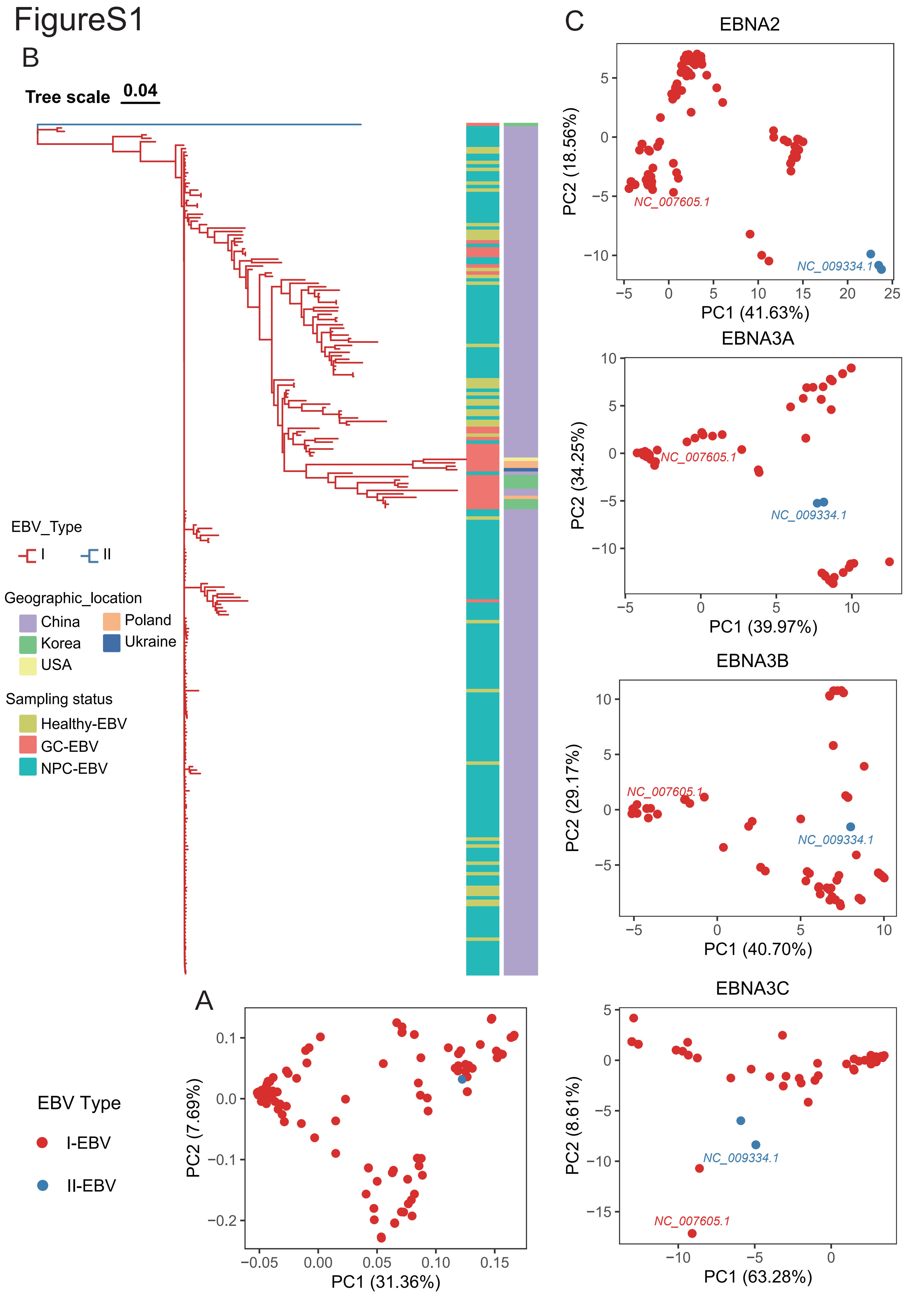
FigureS1
B
Tree scale 0.04
EBV_Type
I
II
Geographic_location
China
Poland
Korea
Ukraine
USA
Sampling status
Healthy-EBV
GC-EBV
NPC-EBV
A
EBV Type
I-EBV
II-EBV
PC1 (31.36%)
PC2 (7.69%)
C
EBNA2
PC1 (41.63%)
PC2 (18.56%)
NC_007605.1
NC_009334.1
EBNA3A
PC1 (39.97%)
PC2 (34.25%)
NC_007605.1
NC_009334.1
EBNA3B
PC1 (40.70%)
PC2 (29.17%)
NC_007605.1
NC_009334.1
EBNA3C
PC1 (63.28%)
PC2 (8.61%)
NC_009334.1
NC_007605.1

FigureS2

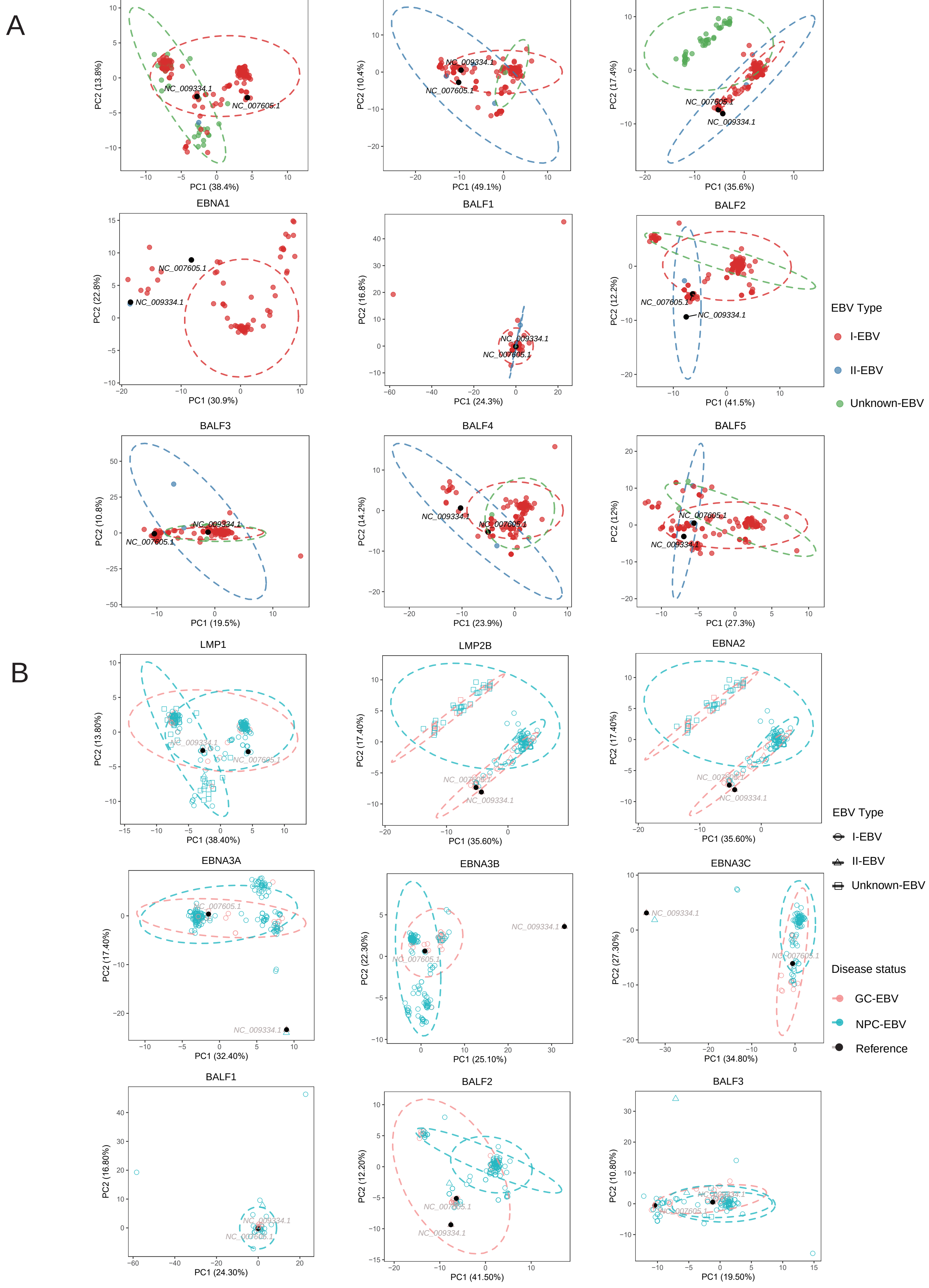
A
LMP1
LMP2A
LMP2B
EBNA1
BALF1
BALF2
BALF3
BALF4
BALF5
PC1 (38.4%)
PC2 (13.8%)
PC1 (49.1%)
PC2 (10.4%)
PC1 (35.6%)
PC2 (17.4%)
PC1 (30.9%)
PC2 (22.8%)
PC1 (24.3%)
PC2 (16.8%)
PC1 (41.5%)
PC2 (12.2%)
PC1 (19.5%)
PC2 (10.8%)
PC1 (23.9%)
PC2 (14.2%)
PC1 (27.3%)
PC2 (12%)
NC_009334.1
NC_007605.1
EBV Type
I-EBV
II-EBV
Unknown-EBV
B
LMP1
LMP2B
EBNA2
EBNA3A
EBNA3B
EBNA3C
BALF1
BALF2
BALF3
PC1 (38.40%)
PC2 (13.80%)
PC1 (35.60%)
PC2 (17.40%)
PC1 (32.40%)
PC2 (17.40%)
PC1 (25.10%)
PC2 (22.30%)
PC1 (34.80%)
PC2 (27.30%)
PC1 (24.30%)
PC2 (16.80%)
PC1 (41.50%)
PC2 (12.20%)
PC1 (19.50%)
PC2 (10.80%)
EBV Type
I-EBV
II-EBV
Unknown-EBV
Disease status
GC-EBV
NPC-EBV
Reference

FigureS3

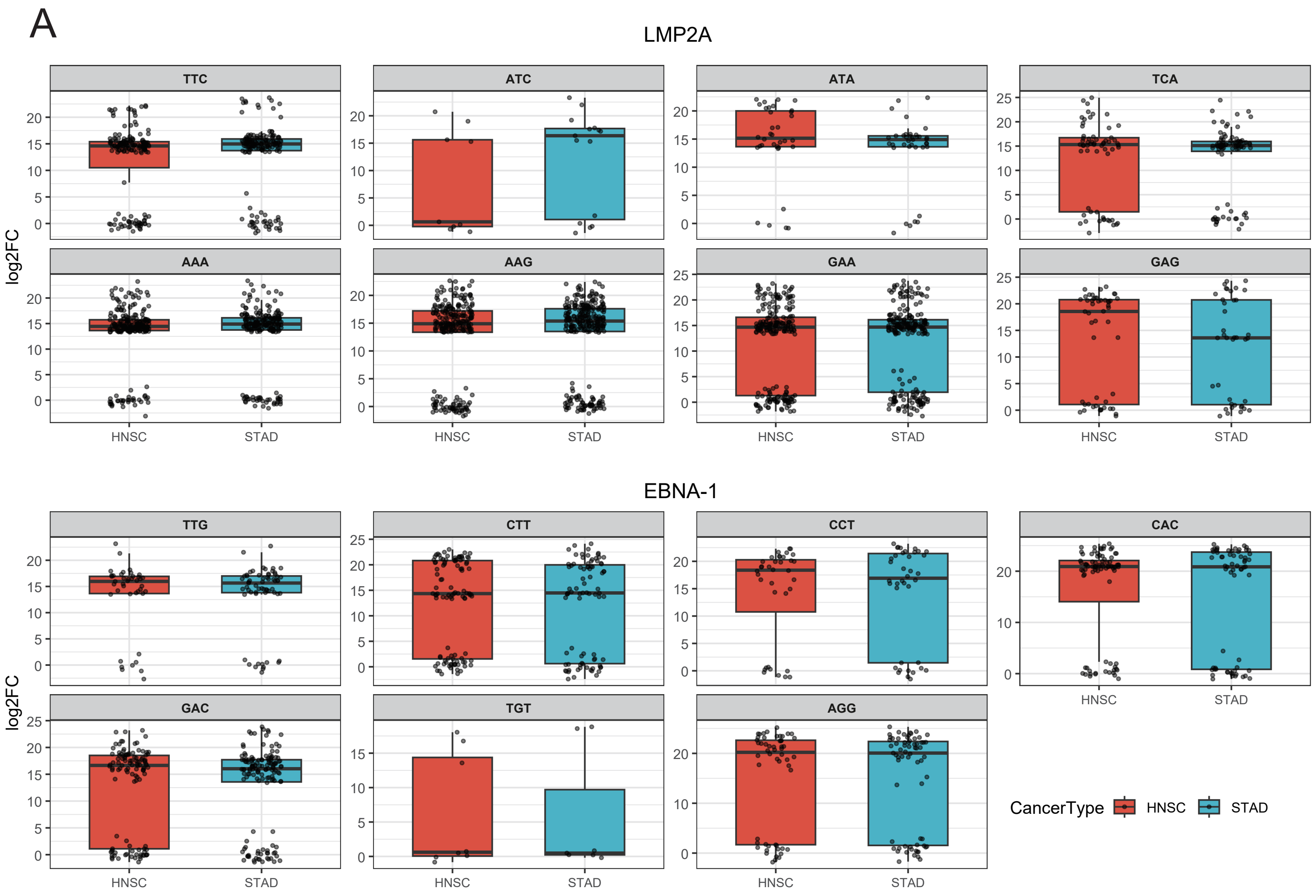
A
LMP2A
TTC
ATC
ATA
TCA
AAA
AAG
GAA
GAG
log2FC
HNSC
STAD
EBNA-1
TTG
CTT
CCT
CAC
GAC
TGT
AGG
CancerType
HNSC
STAD

FigureS4

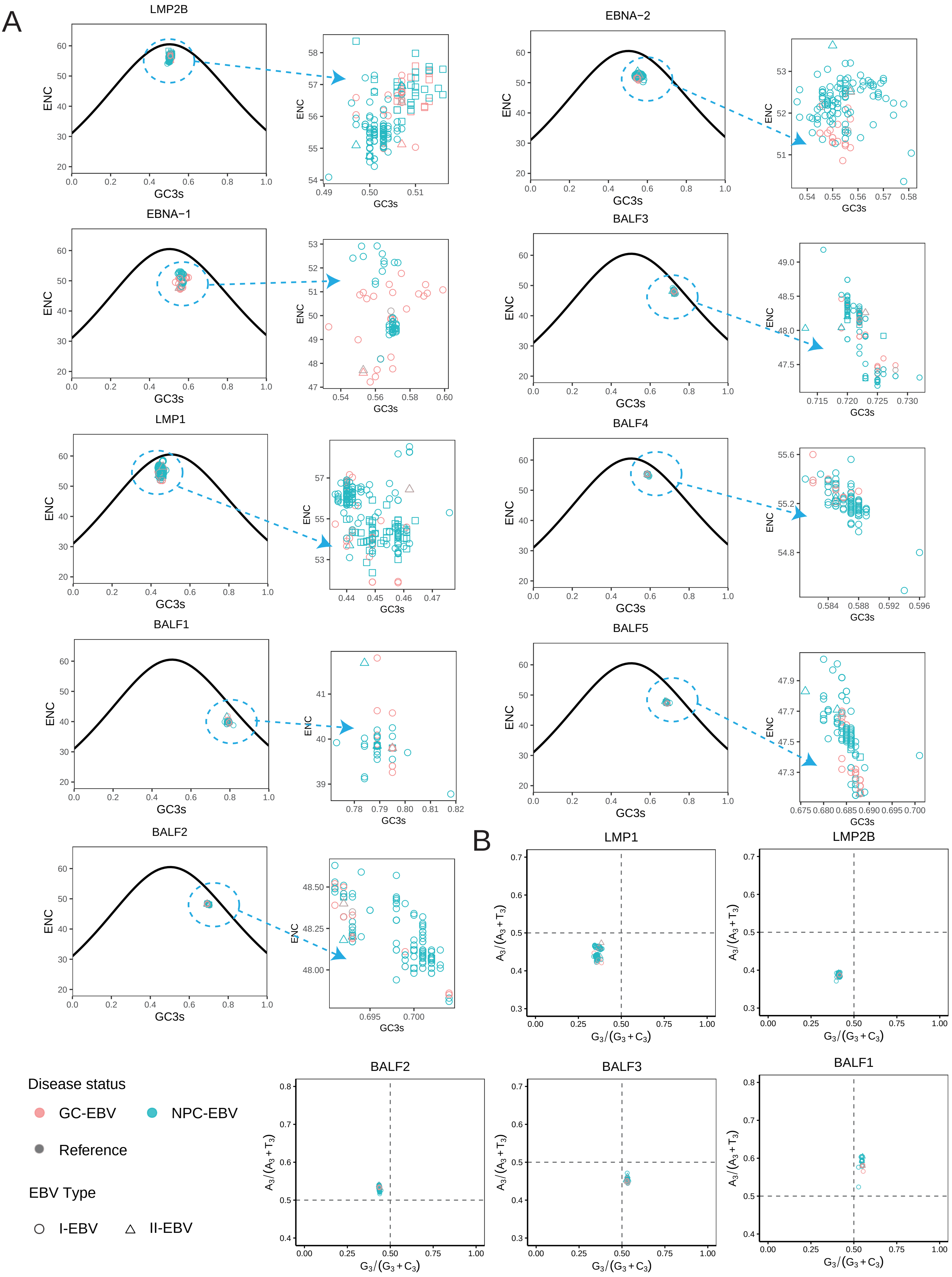

A
LMP2B
EBNA−2
EBNA−1
BALF3
LMP1
BALF4
BALF1
BALF5
BALF2
ENC
GC3s
B
LMP1
LMP2B
BALF2
BALF3
BALF1
$A_3/(A_3+T_3)$
$G_3/(G_3+C_3)$
Disease status
GC-EBV
NPC-EBV
Reference
EBV Type
I-EBV
II-EBV

FigureS5

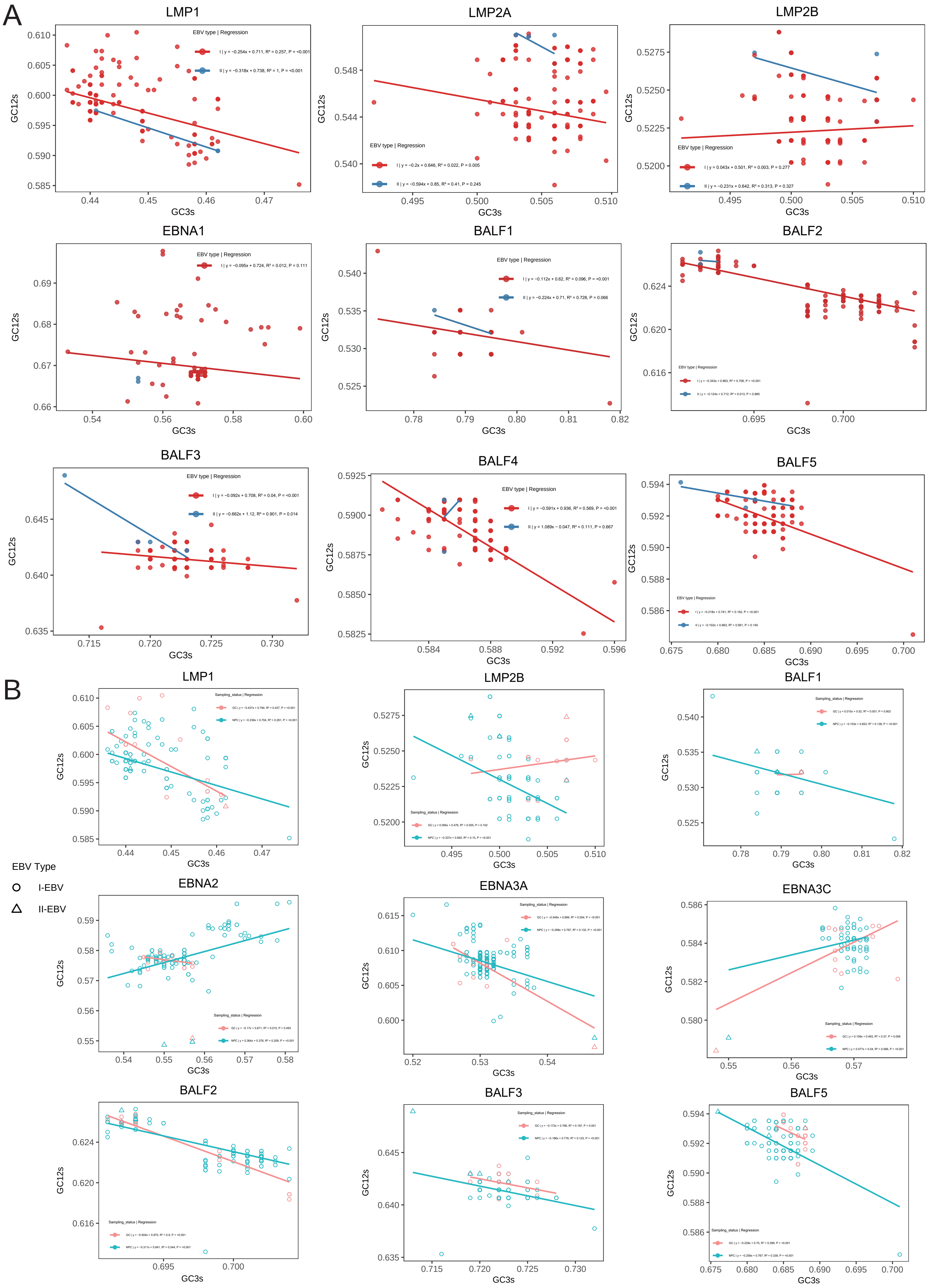

A
LMP1
LMP2A
LMP2B
EBNA1
BALF1
BALF2
BALF3
BALF4
BALF5
GC12s
GC3s
EBV type | Regression
B
LMP1
LMP2B
BALF1
EBNA2
EBNA3A
EBNA3C
BALF2
BALF3
BALF5
EBV Type
I-EBV
II-EBV
Sampling_status | Regression

FigureS6

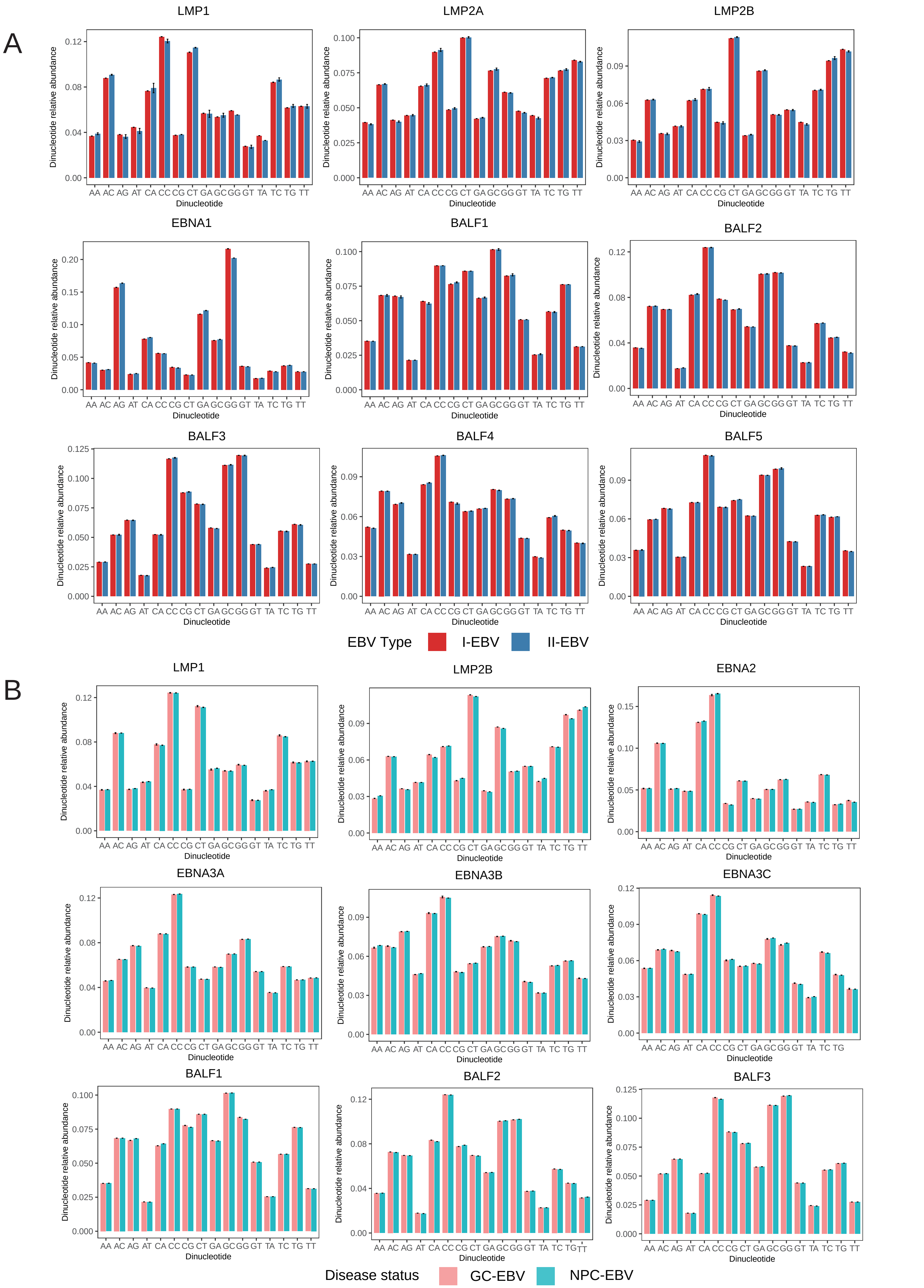
A
LMP1
LMP2A
LMP2B
EBNA1
BALF1
BALF2
BALF3
BALF4
BALF5
Dinucleotide relative abundance
Dinucleotide
EBV Type
I-EBV
II-EBV
B
LMP1
LMP2B
EBNA2
EBNA3A
EBNA3B
EBNA3C
BALF1
BALF2
BALF3
Dinucleotide relative abundance
Dinucleotide
Disease status
GC-EBV
NPC-EBV
